# Supplementary material for: Splice-Junction-Based Mapping of Alternative Isoforms in the Human Proteome
Source: Cell Rep. Author manuscript; Available in PMC 2020 Jan 15. (PMC6961840; doi:10.1016/j.celrep.2019.11.026)
Supplement: 2 [file NIHMS1546469-supplement-2.zip › DF1/PXD006675/LeftCoronaryArtery/LeftCoronaryArtery_1_COL6A1_IVTFSSPADITILLDGSASVGSHNFDTTK.pdf]

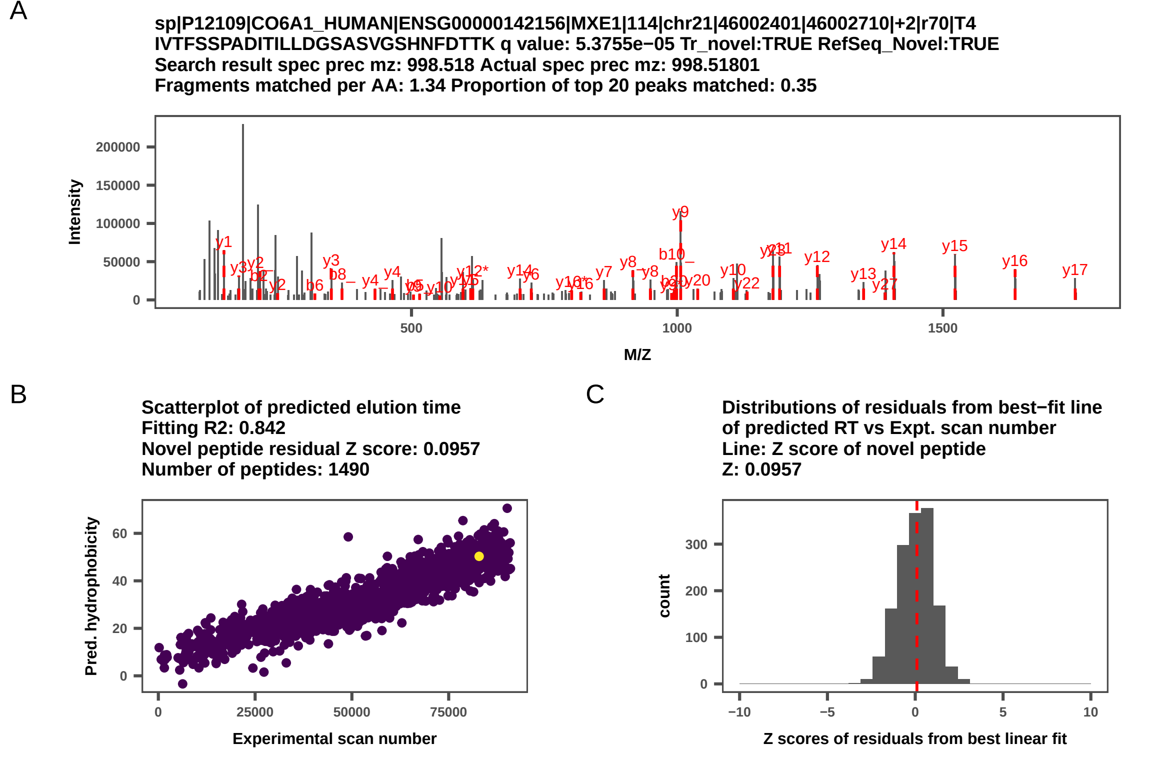
A
sp|P12109|CO6A1_HUMAN|ENSG00000142156|MXE1|114|chr21|46002401|46002710|+2|r70|T4
IVTFSSPADITILLDGSASVGSHNFDTTK q value: 5.3755e−05 Tr_novel:TRUE RefSeq_Novel:TRUE
Search result spec prec mz: 998.518 Actual spec prec mz: 998.51801
Fragments matched per AA: 1.34 Proportion of top 20 peaks matched: 0.35
Intensity
200000
150000
100000
50000
0
500
1000
1500
M/Z
B
Scatterplot of predicted elution time
Fitting R2: 0.842
Novel peptide residual Z score: 0.0957
Number of peptides: 1490
Pred. hydrophobicity
60
40
20
0
25000
50000
75000
Experimental scan number
C
Distributions of residuals from best−fit line
of predicted RT vs Expt. scan number
Line: Z score of novel peptide
Z: 0.0957
count
300
200
100
0
−10
−5
0
5
10
Z scores of residuals from best linear fit
